# Electronic characterization of redox (non)-innocent Fe_2_S_2_ reference systems: a multi K-edge X-ray spectroscopic study†

**DOI:** 10.1039/c9ra08903a

**Published:** 2020-01-02

**Authors:** J. P. H. Oudsen, B. Venderbosch, T. J. Korstanje, M. Tromp

**Affiliations:** Sustainable Materials Characterization, Van't Hoff Institute for Molecular Sciences, University of Amsterdam Science Park 904 1098 XH Amsterdam The Netherlands; Materials Chemistry, Zernike Institute for Advanced Materials Nijenborgh 4 9747AG Groningen The Netherlands Moniek.Tromp@rug.nl

## Abstract

Di-iron dithiolate hydrogenase model complexes are promising systems for electrocatalytic production of dihydrogen and have therefore been spectroscopically and theoretically investigated in this study. The direct effect of ligand substitution on the redox activity of the complex is examined. In order to understand and eventually optimize such systems, we characterised both metal and ligand in detail, using element specific X-ray absorption Fe- and S-K edge XAS. The (electronic) structure of three different [Fe_2_S_2_] hydrogenase systems in their non-reduced state was investigated. The effect of one- and two-electron reduction on the (electronic) structure was subsequently investigated. The S K-edge XAS spectra proved to be sensitive to delocalization of the electron density into the aromatic ring. The earlier postulated charge and spin localization in these complexes could now be measured directly using XANES. Moreover, the electron density (from S K-edge XANES) could be directly correlated to the Fe–CO bond length (from Fe K-edge EXAFS), which are in turn both related to the reported catalytic activity of these complexes. The delocalization of the electron density into the conjugated π-system of the aromatic moieties lowers the basicity of the diiron core and since protonation occurs at the diiron (as a rate determining step), lowering the basicity decreases the extent of protonation and consequently the catalytic activity.

## Abstract 2

Di-iron dithiolate hydrogenase model complexes are promising systems for electrocatalytic production of dihydrogen and have therefore been spectroscopically and theoretically investigated in this study.

## Graphical abstract

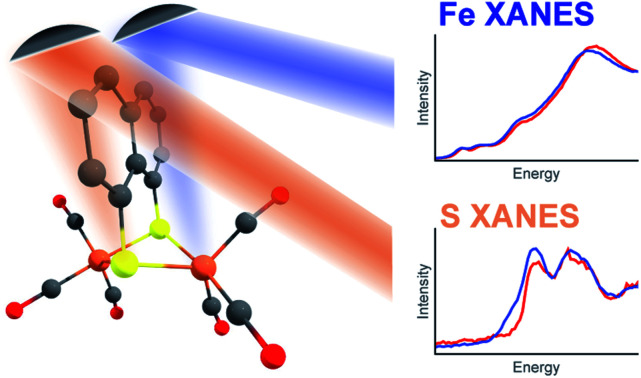

## 1. Introduction

Despite the variety of Fe_2_S_2_ hydrogenase mimics synthesized and evaluated for hydrogen evolution, a greater understanding of the role of the subsequent ligand is required. In general, the spectator role or ‘innocence’ of the ligand has proven inaccurate for more and more ligands and care has to be taken in judging the ligand's role and capabilities,^1,2^ especially when trying to explain the performance and reaction mechanisms of organometallic complexes.^3^ Thus far, in our group, doubly reduced [Fe_2_(bdt)CO_6_]^2−^[1]^2−^ species have been characterized structurally and electronically by Fe K-edge XAS spectroscopy, showing the rupture of an iron–sulfur bond, caused by the potential inversion.^4^ Time resolved infrared spectroscopy (TRIR) enabled the characterization of reduced intermediate structures, Mirmohades *et al.*^5^ for example showed Fe–S bond rupture after the first reduction by laser quench methods. A variety of functionalized 1,8-dithiolene diiron complexes were synthesized and electrochemically characterized. The authors postulated both events to reduce Fe^1^–Fe^1^ towards monomeric Fe^0^–Fe^0^, although possible ligand reduction could not be excluded.^6^ This is in contrast to investigations by the group of X. Liu, where ferrocene functionalized 1,8-dithiol-naphthalene hydrogenase mimics were postulated to form a dimer with a tetra-iron core,^7^ also observed in alkyl bridgehead structures.^8,9^ In both systems, the reduction event is described to take place at the metal. Hence, the use of naphthalene mono imide (NMI) substituted hydrogenase system^10^ showed for the first time an ultrafast electron transfer process between excited ZnTPP and the ligand of a Fe_2_S_2_ H_2_-ase model in a self-assembled system. The electronic structure of [3]^1−^ reveals that the NMI group is non-innocent during this first reduction, which explains the low activity in photo-driven hydrogen production.^10^ This ligand based reduction was revealed by IR spectro-electrochemistry (IR SEC), EPR spectroscopy in combination with DFT calculations.^9^ Using combined S K-edge and Fe K-edge XAS, we seek to describe and understand the effect of ligand environment in the H-cluster mimics in much more detail.

**Scheme 1:**
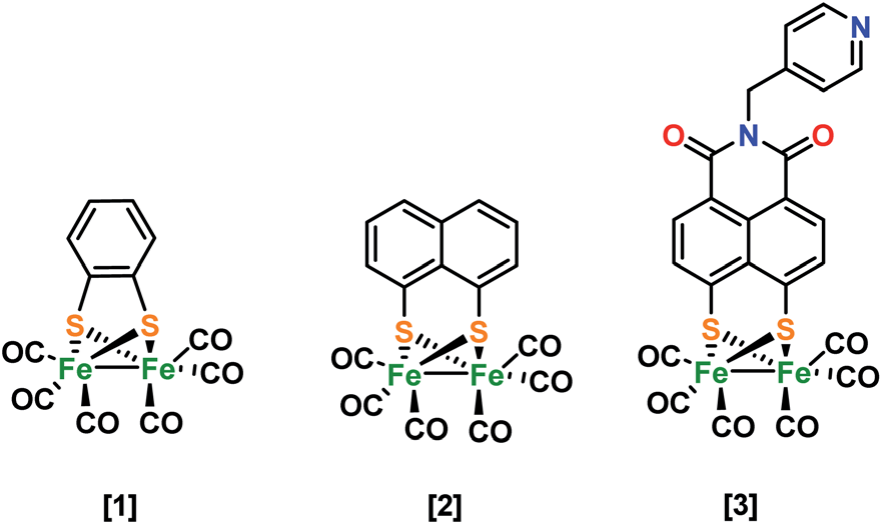
Hydrogenase mimic structures investigated in this study.

The group of Solomon pioneered in this field, using S K-edge spectroscopy to investigate sulfur covalency in [2Fe–2S] sites of model complexes where sulfide were distinguished from the thiolate pre-edge due to differences in effective nuclear charge.^11^ Earlier investigations by the same group investigated the active site of the non-heme iron enzyme nitrile hydratase (NHase) using S K-edge XAS. They evaluated the effects of metal coordination to the S, protonation/metal coordination to the O of RSO^−^, and protonation of RSO_2_^−^. DFT calculations (solely ground state) were used to assign the significant shifts between the different species. The group of Hedman and Solomon has shown by a combination of Cu K- and L-edge and S K-edge XAS a metal-based oxidation to occur upon one-electron oxidation.^12^ The observed pre-edge and edge energy shifts observed in these XAS spectra were supported by DFT calculations.^12^ TD-DFT calculations have been performed on geometrically different compounds with identical functional groups by the group of Hedman. They demonstrated the strength of combining theory and spectroscopy as they could clearly distinguish between functional groups based on the mixing of atomic orbitals and the energies of molecular orbitals leading to changes in the S K-edge XANES spectra.^13^ The group of De Beer has presented Fe K-, S K-edge XAS in combination with Fe Kβ XES data on a series of Fe_2_S_2_ complexes, which span three redox levels.^14^ The data displayed a detailed picture of electronic structures. By the use of Fe Kβ XES and K-edge XAS they emphasize that “fingerprints” regarding oxidation states should be treated with caution.^14^ The series of [Fe_2_S_2_] mimics in this study are proposed to have very different charge and spin localization in the complexes. Next to envisioned differences in electronic structure we were able to also measure corresponding Fe–S and/or Fe–Fe bond elongations directly (Scheme 1).

## 2. Experimental

### 2.1 General procedures

Starting materials were obtained commercially or prepared and purified according to the references given below. All solvents were dried by using standard procedures.^15^ Ligand (L) 1,8-naphthalic anhydride-4,5-disulfide was prepared *via* literature procedure.^16^ All air-sensitive materials were manipulated using standard Schlenk techniques or by the use of a glovebox. ^1^H and ^13^C NMR spectra were recorded on a Bruker AVANCE 400 MHz spectrometer. IR spectra were recorded on a Nicolet Nexus FT-IR spectrometer.

### 2.2 Sample preparation

#### [Fe_2_(bdt)(CO)_6_] [1]

A modified literature procedure^17^ was used. 4.3 g (8.5 mmol) Fe_3_(CO)_12_ was treated with 1.20 g (8.5 mmol) benzene-1,2-dithiol in THF and refluxed for exactly two hours. The resulting red solution was directly filtered over cotton. Unreacted Fe(CO)_5_ was evaporated by rotary evaporation as a yellow solution. Silica column chromatography was conducted using petroleum ether/pentane (40/60) as eluent. Only the red band was collected and further dried *in vacuo*, resulting in red crystalline powder in a 76% yield. ^1^H NMR (300 MHz, CDCl_3_) *δ* 7.16 (dd, *J* = 5.5, 3.2 Hz, 2H), 6.66 (dd, *J* = 5.5, 3.2 Hz, 2H) ppm. ^13^C{^1^H} NMR (300 MHz, CDCl_3_): *δ* = 207.48 (s, CO), 147.45 (s, SC), 127.96 (s, SCCH), 126.80 (s, SCCHCH) ppm. IR (CO) (MeCN) (cm^−1^): 2078 (m), 2043 (s), 2002 (s).

#### [Fe_2_(bdt)(μ-CO)(CO)_5_]^2−^[1]^2−^

2.1 eq. of 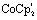 (5 mM) was added to [Fe_2_(bdt)(CO)_6_] (5.0 mM) in acetonitrile under nitrogen or argon atmosphere. IR (CO) (MeCN) (cm^−1^): 1963 (s), 1914 (s), 1881 (w), 1863 (m), 1842 (w), 1688 (b).

#### Napthalene-1,8-dithiolate [2a]

720 mg (2.3 mmol) of sulfur and 570 mg (2.3 mmol) of sodium was refluxed in 40 mL of DMF under nitrogen atmosphere. 1.50 g of 1,8 di-bromonaphthalene was added upon the solution turned dark blue over time. The mixture was refluxed for 24 h and afterwards cooled down to room temperature. To the solution was added 50 mL of water followed by 100 mL of toluene. The organic phase was extracted and washed with water (2 × 50 mL) using a separation funnel. The solution was evaporated using a rotary evaporator, leaving an orange oil. Recrystallization in hexane at −20 °C overnight yielded orange/red spiked crystals. ^1^H-NMR (400 MHz, CD_2_Cl_2_) *δ* 8.33 (dd, *J* = 7.3, 1.2 Hz, 2H), 8.10 (dd, *J* = 8.2, 1.2 Hz, 2H), 7.49 (t, *J* = 7.8 Hz, 2H).

#### [Fe_2_(naph)(CO)_6_] [2]

Naphthalene-1,8-dithiol [2a] (1.15 g, 6.00 mmol) was dissolved in THF (150 mL). Dissolved [Fe_2_(CO)_9_] (3.02 g, 6.00 mmol), in THF (20 mL) and added dropwise to the ligand solution. The mixture was refluxed for 45 min as it turned from green to red. Finally, the solution was allowed to cool to room temperature, filtered, and concentrated to dryness. The residue was recrystallized in hexane (20 mL), affording microcrystalline product [2] (1.4 g, 50%). ^1^H-NMR (400 MHz, CDCl_3_) *δ* 8.28 (d, *J* = 7.1 Hz, 2H), 8.02 (d, *J* = 8.2 Hz, 2H), 7.44 (t, *J* = 7.5 Hz, 2H) ppm. IR (CO) (MeCN) (cm^−1^): 2075 (m), 2041 (s), 2000 (br, m).

#### [Fe_2_(naph)(CO)_6_]^1−^[2]^1−^

1.05 eq. of 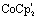 (5 mM), added to [Fe_2_(naph)(CO)_6_] (5.0 mM) in acetonitrile under nitrogen or argon atmosphere. IR (CO) (MeCN) (cm^−1^): 2008 (m, 1950 (s), 1915 (br, m)).

#### [Fe_2_(naph)(CO)_6_]^2−^[2]^2−^

2.1 eq. of 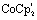 (5 mM) was added to [Fe_2_(naph)(CO)_6_] (5.0 mM) in acetonitrile under nitrogen or argon atmosphere. IR (CO) (MeCN) (cm^−1^): 1920 (s), 1890 (s), 1864 (br, m), 1805 (br, m).

#### μ-PyCH_2_-NMI-S_2_[3a]

1,8-Naphthalic anhydride-4,5-disulfide (260 mg, 1.15 mmol) was dissolved in degassed 2-methoxyethanol and 0.7 mL (740 mg) of 4-aminomethylpyridine was added. The solution was refluxed for 48 hours under nitrogen atmosphere. The solution was cooled down and placed at −20 °C for 48 hours, after which golden crystals were obtained and further dried *in vacuo* (165 mg) in 45% yield. ^1^H-NMR (400 MHz, CDCl_3_) *δ* 8.54 (d, *J* = 6.1 Hz 2H), 8.47 (d, *J* = 8.1 Hz, 2H), 7.52 (d, *J* = 8.1 Hz,2H), 7.36 (d, *J* = 5.7 Hz, 2H), 5.39 (s, 2H) ppm.

#### [μ-(PyCH_2_-NMI-S_2_)Fe_2_(CO)_6_] [3]

Ligand [3a] (300 mg, 0.86 mmol) was dissolved in dry THF after which Fe_3_CO_12_ (432 mg, 0.86 mmol) was dissolved in 10 mL THF and added to the reaction mixture. After stirring for 1.5 hour at room temperature, the reaction mixture turned from green to red and was filtered over celite and further dried *in vacuo*. The product was purified by column chromatography on silica gel with CH_2_Cl_2_ and a drop of methanol. The product was obtained as pure orange powder in 46% yield. ^1^H-NMR (400 MHz, CDCl_3_) *δ* 8.56 (2H), 8.44 (2H), 7.54 (2H), 7.37 (2H), 5.34 (2H) ppm. IR (CO) (MeCN) (cm^−1^): 2080 (m), 2045 (s), 2007 (s).

#### [μ-(PyCH_2_-NMI-S_2_)Fe_2_(CO)_6_]^1−^[2]^1−^

1.05 eq. of 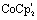 (5 mM), added to [μ-(PyCH_2_-NMI-S_2_)Fe_2_(CO)_6_] (5.0 mM) in acetonitrile under nitrogen or argon atmosphere IR (CO) (MeCN) (cm^−1^): 2050 (m), 2018 (s), 1979 (br, m).

### 2.3 Infra-red spectro-electrochemistry

Electrochemical experiments were performed in an optically transparent thin-layer (200 μm) electrochemical (OTTLE)^18^ cell equipped with CaF_2_ optical windows and a platinum minigrid working electrode. The difference absorbance IR spectra were recorded on a Nicolet Nexus FT-IR spectrometer. The cyclic voltammetry scanning process (*v* = 0.5 mV s^−1^) was controlled by a PGSTAT (Eco-Chemie) potentiostat. The measurements were performed on 5.0 mM complexes in acetonitrile containing 0.1 M *n*Bu_4_NPF_6_ as a supporting electrolyte.

### 2.4 Fe K-edge X-ray absorption spectroscopy

Fe K-edge (7112 eV) X-ray absorption measurements were performed at beamline B18 of the Diamond Light Source (Didcot, UK) using a Si (111) crystal monochromator. Solutions were measured in fluorescence mode, using a germanium 9 element detector, or in transmission using ionisation chambers. The measurements were performed on 5.0 mM complexes in acetonitrile. Samples were prepared in peek cells in a glovebox under argon atmosphere and were sealed with 8 microns thick kapton foil as X-ray window. The energy was calibrated by defining the first derivative peak of the Fe foil spectrum to be 7112.0 eV. Data processing and analysis was conducted with Athena and Artemis (Demeter software package).^19^

### 2.5 S K-edge X-ray absorption spectroscopy

S K-edge (2472 eV) X-ray absorption measurements were performed at beamline B18 of the Diamond Light source (Didcot, UK) using a Si (111) crystal monochromator. The cell was mounted in a large vacuum chamber. Solutions were always measured under high vacuum (to prevent strong absorption of X-rays by air) and measured in fluorescence mode, using a germanium 9 element detector. The measurements were performed on 5.0 mM complexes in acetonitrile. Samples were prepared in peek cells in a glovebox under argon atmosphere and were sealed with 8 microns thick kapton foil as X-ray window. Energy calibration was performed with FeS_2_ for which the peak of the white line was assigned to 2471.1 eV. In order to exclude beam induced changes, we monitored beam irradiation effects by 10 subsequent quick scans (2 min per scan), with a focused beam on complex [1]. No changes in the XANES spectra were observed. Additionally, Fe K-edge XANES was always measured before and after every S K-edge XANES experiment to check the stability of the complexes in solution. After each experiment, the sample was inspected for beam damage by eye. As such we ensure that reported spectral changes are in fact chemistry and not beam induced. Data processing and analysis was conducted with Athena (Demeter software package).^19^

### 2.6 Computational details

All geometry optimizations and IR simulations were conducted using ADF^20^ with the TZ2P basis set and BP86 (ref. 21) or OPBE^22^ functional. TD-DFT XANES calculations were performed with a 50 Davidson excitation restriction window where only quadrupole- and dipole-allowed transitions are selected from the Fe 1s orbitals.^23^ In all TD-DFT calculations, the B3LYP – d3 functional and a QZ4P Slater-type basis set was applied.^24^ The intensities include second-order contributions due to the magnetic-dipole and electric–quadrupole transition moments.^25^ The spectra were shifted by 151.0 eV in case of iron and 56.0 eV in case of sulfur for comparison to experiment.^26^ These shifts are chosen such that the energy of the first peak in the calculated spectrum agrees with the first peak in the experimental spectrum. While these shifts are rather large, they do not affect the relative position of the peaks.

## 3. Results and discussion

### 3.1 IR spectro-electrochemistry

The spectro-electrochemistry of complex [1] was already discussed in our previous work.^4^ In here, four main CO vibrational modes in the IR region, *i.e.* at 2078 cm^−1^, 2044 cm^−1^, and two signals near 2001 cm^−1^, underwent a blue shift towards 1961 cm^−1^, 1914 cm^−1^ and 1862 cm^−1^. An additional signal at 1682 cm^−1^ was assigned to a bridging carbonyl, showing the formation of [1]^2−^. The reported DFT calculations were in excellent agreement with these experimental results. By applying a potential over a solution of [2], the first reduced state [2]^1−^ could be characterized with a reduction potential of roughly −1.6 eV, in correspondence with literature.^6,10^ The vibrational bands originally present at 2074 cm^−1^, 2038 cm^−1^ and 1999 cm^−1^ also here underwent a blue shift towards 2008 cm^−1^ 1950 cm^−1^ and 1910 cm^−1^, in correspondence with literature.^10^ Moreover, our computational data (Fig. S1†) again agree nicely. Upon increase of the potential, near −1.95 eV new strong bands at 1910, 1880 cm^−1^ and broad bands between 1850 and 1800 cm^−1^ (Fig. S2†) were observed. DFT calculations reproduce these experiments well, predicting bands at 1910 cm^−1^, 1895 cm^−1^ and two relatively sharp signals between 1850 and 1800 cm^−1^. This would imply that all carbonyls are ligated in a linear fashion towards an even more reduced iron core. Steady state infrared spectra are recorded for [3] and [3]^1−^ (Fig. S3†) and showed identical values compared to results obtained by Ping *et al.*^10^ The neutral state has three signals at 2080 cm^−1^, 2046 cm^−1^ and 2007 cm^−1^, while the reduced state displays signals at 2054 cm^−1^, 2018 cm^−1^ and 1976 cm^−1^. Next to the metal-carbonyls, two additional weak bands are shifted from 1706 cm^−1^ and 1666 cm^−1^ towards 1629 cm^−1^ and 1583 cm^−1^, highlighting the largely delocalized spin density upon the ligand system.

### 3.2 Fe K-edge EXAFS

**Fig. 1:**
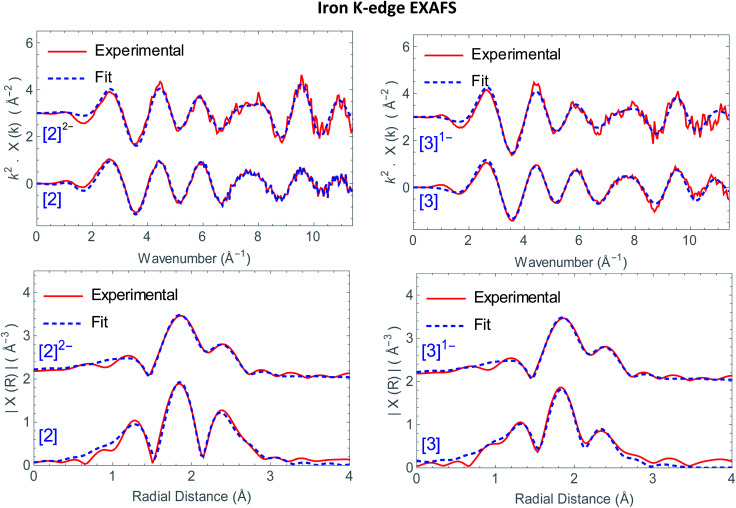
(Top) *k*^2^-weighted Fe K-edge EXAFS data of [2] and [2]^2−^ (left), [3] and [3]^1−^ (right). (Bottom) *k*^2^-weighted Fourier transforms of the EXAFS data of [2] and [2]^2−^ (left), [3] and [3]^1–^ (right). In all plots the data are represented by the solid lines (red), whereas the corresponding fits are the dotted lines (blue).

Structural information on the precursors [2] and [3] together with reduced species [2]^1−^, [2]^2−^ and [3]^1−^ was obtained *via* Fe K-edge EXAFS analysis. An overview of all the obtained Fe K edge EXAFS data of both precursors and final reduced species is presented in Fig. 1. The structural information for [1] and its chemically reduced analogue [1]^2−^ have been extensively discussed in our previous work.^4^ We characterized the di-anionic structure as an open structure in which one Fe–S bond was broken in combination with the formation of a new bridging carbonyl between both iron centres. Precursors [2] and [3] were measured in acetonitrile and require four shells to obtain a satisfactory fit for the data. The first shell is a carbon shell containing three atoms at a distance of ∼1.80 Å. The second shell is a sulfur shell, containing two atoms at 2.26 Å. The third shell is an iron shell containing one atom at a distance of 2.43(4) Å in case of [2] and 2.539(2) Å in case of [3]. The final oxygen shell has a coordination number of three at ∼2.93 Å. Additional multiple scattering pathways, *i.e.* single forward Fe–CO and double forward Fe–COC, were analysed for both structures. In order to reduce the amount of parameters used in the fit, the Fe–CO and Fe–COC multiple scattering pathways were constrained by applying the parameters used for the single scattering contributions of the carbon and oxygen shell, assuming perfectly linear Fe–CO coordination geometry.

The *k*^2^-weighted Fourier transform of [2]^1−^ in acetonitrile is shown in Fig. S4.† The obtained EXAFS parameters and comparison towards a DFT optimized structure are given in Table 1. Also here, four shells are used to obtain a reliable fit for the data. The first shell, still has three carbon atoms at a similar bond distance of 1.806(8) Å compared to [2]. The second shell of two sulfur atoms is 2.27(1) Å. Whereas the third shell with one iron atom, is elongated extensively towards 3.12(4) Å, in line with DFT (2.85 Å).‡‡The use of 1.05 eq. 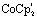 results in partial formation of [1]^2−^ explaining the longer Fe–Fe bond distance in [1]. The final oxygen shell consists of three atoms and remains at a distance of ∼2.95 Å. The fact that [2]^1−^ has three identical Fe–CO bond distances and an identical number of Fe–CO and Fe–COC multiple scattering pathways compared to [2] proves that no bridged carbonyl species (Fe–μCO) are present in the bulk solution.

**Table 1:** Fe K-edge EXAFS fitting parameters for [2], [2]^1−^, [2]^2−^, [3] and [3]^1−^ where *N* = coordination number, *σ*^2^ = Debye Waller factor [Å^−2^], *R* = fitted bond length [Å]

Sample	Shell	*N*	*σ* ^2^ (Å^2^)	*R*_DFT_ (Å)	*R*_fit_ (Å)
[2]^a^	Fe–C	3	0.004(2)	1.79	1.80(2)
	Fe–S	2	0.002(3)	2.27	2.28(2)
	Fe–Fe	1	0.004(5)	2.54	2.43(4)
	Fe–O	3	0.001(3)	2.93	2.95(1)
	Fe–CO	6	0.002(2)	2.93	2.95(1)
	Fe–COC	3	0.004(4)	2.93	2.95(1)
[2]^1−^b	Fe–C	3	0.004(1)	1.80	1.806(8)
	Fe–S	2	0.0018(7)	2.33	2.27(1)
	Fe–Fe	1	0.007(5)	2.85	3.12(4)
	Fe–O	3	0.011(4)	2.93	2.95(1)
	Fe–CO	6	0.008(4)	2.93	2.95(1)
	Fe–COC	3	0.008(4)	2.93	2.95(1)
[2]^2−^c	Fe–C	3	0.006(2)	1.76	1.80(1)
	Fe–S	2	0.0022(7)	2.36	2.29(2)
	Fe–Fe	1	0.01(1)	3.46	3.53(8)
	Fe–O	3	0.012(3)	2.87*	2.83(1)
	Fe–CO	6	0.010(3)	2.87*	2.83(1)
	Fe–COC	3	0.010(3)	2.87*	2.84(1)
[3]^d^	Fe–C	3	0.003(2)	1.80	1.82(1)
	Fe–S	2	0.003(7)	2.26	2.253(8)
	Fe–Fe	1	0.008(1)	2.54	2.539(2)
	Fe–O	3	0.005(3)	2.94	2.989(2)
	Fe–CO	6	0.005(4)	2.94	2.989(8)
	Fe–COC	3	0.010(4)	2.94	2.989(8)
[3]^1−^e	Fe–C	3	0.007(2)	1.77	1.79(2)
	Fe–S	2	0.004(2)	2.29	2.27(1)
	Fe–Fe	1	0.008(7)	2.55	2.51(4)
	Fe–O	3	0.003(4)	2.93	2.95(2)
	Fe–CO	6	0.005(5)	2.93	2.95(2)
	Fe–COC	3	0.010(5)	2.93	2.95(2)

a *k* range = 3–11.4 Å, *R* range = 1–3.5 Å; *k*-weighted fit = 1,2,3 *E*_0_ = −3.4 eV, *S*_0_^2^ = 0.90. *R*-factor fit: 0.021.

b *k* range = 3–10.5 Å, *R* range = 1–4.0 Å; *k*-weighted fit = 1,2,3 *E*_0_ = 1.16 eV, *S*_0_^2^ = 0.90. *R*-factor fit: 0.015.

c *k* range = 3–11.2 Å, *R* range = 1–3.5 Å; *k*-weighted fit = 1,2,3 *E*_0_ = −2(1) eV, *S*_0_^2^ = 0.90. *R*-factor fit: 0.018.

d *k* range = 3–12.0 Å, *R* range = 1–3.5 Å; *k*-weighted fit = 1,2,3 *E*_0_ = 0.59 eV, *S*_0_^2^ = 0.90. *R*-factor fit: 0.020.

e *k* range = 2–11.8 Å, *R* range = 1–3.5 Å; *k*-weighted fit = 1,2,3 *E*_0_ = 1(1) eV, *S*_0_^2^ = 0.90. *R*-factor fit: 0.023. *The Fe–CO bond angle changes from almost 180° towards 172° shortening the overall scattering pathway and the corresponding Fe–CO distance.

The *k*^2^-weighted Fourier transform of [2]^2−^ in acetonitrile is shown in Fig. 1. The obtained EXAFS parameters and comparison towards a DFT optimized structure is given in Table 1. Although the first carbon shell of three atoms is still at a similar distance of 1.80(1) Å, the second shell with two sulfur atoms underwent a small, but further elongation towards 2.29(2) Å. In case of the iron shell, an elongation towards 3.53(8) Å was observed. Finally, the Fe–COs are shortened towards 2.83(1) Å, but do remain in a linear binding mode based on the single and multiple Fe–CO(C) scattering pathways required for analysis.

This is in good agreement with our spectro-electrochemistry investigations for both reduced species, as only terminal ligated CO signals were observed. The *k*^2^-weighted Fourier transform of [3]^1−^ in acetonitrile is shown in Fig. 1. The obtained EXAFS parameters and comparison towards a DFT optimized structure is given in Table 1. Also here, the first carbon shell of three atoms is at a similar distance of 1.79(1) Å, compared to [3]. The second shell with two sulfur atoms are observed at 2.27(1) Å. Reduction of [2] displayed extensive elongation of the Fe–Fe bond. In case of [3] however, the addition 1.05 eq. 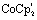 leads to no significant changes in the Fe–Fe bond length. Furthermore, the six Fe–CO bonds are again not affected by the reducing conditions, nor does the Fe–S coordination change.

Based on many electrochemical investigations, the di-iron core is in general known to be capable of accepting one and often two consecutive electrons. In some cases, after accepting the first electron, one of the four Fe–S bonds may de-coordinate to form an entirely new structure.^8,27,28^ Earlier investigations in our group has shown a clear example of this where Fe–S bond breakage even resulted in a potential inversion in [1].^4^ Earlier investigations by G. Qian *et al.*^7^ postulate the formation of a dimer with a tetra-iron core using a ferrocene functionalized 1,8 dithiol-naphthalene hydrogenase mimic. The formation of Fe–S ruptured structures can be disregarded here, as in all cases both bridging sulfur atoms are observed at similar bond distances compared to its neutral state. Furthermore, plausible isomeric structures were also disregarded as in the EXAFS region we only observed an elongation of the Fe–Fe bond and no additional Fe–Fe shells were observed in the experimental R-space.^29^

### 3.3 Fe K-edge XANES

**Fig. 2:**
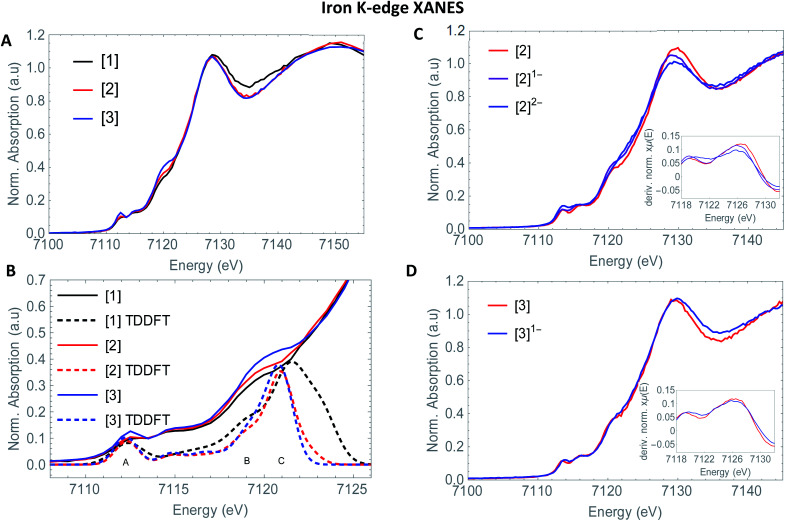
Experimental (solid) and computational (dashed) Fe K edge XANES spectrum of A: [1] (black), [2] (red) and [3] (blue) B: pre-edge region of Fe K-edge XANES spectra of [1] (black), [2] (red) and [3] (blue) with TD-DFT simulated spectra (dashed) C: Fe K-edge XANES spectra of [2] (red) [2]^1−^ (purple) and [2]^2−^ (blue) with the normalized derivative as inset and D: Fe–K edge XANES spectra of [3] (red) and [3]^1−^ (blue) with the normalized derivative as inset.

**Fig. 3:**
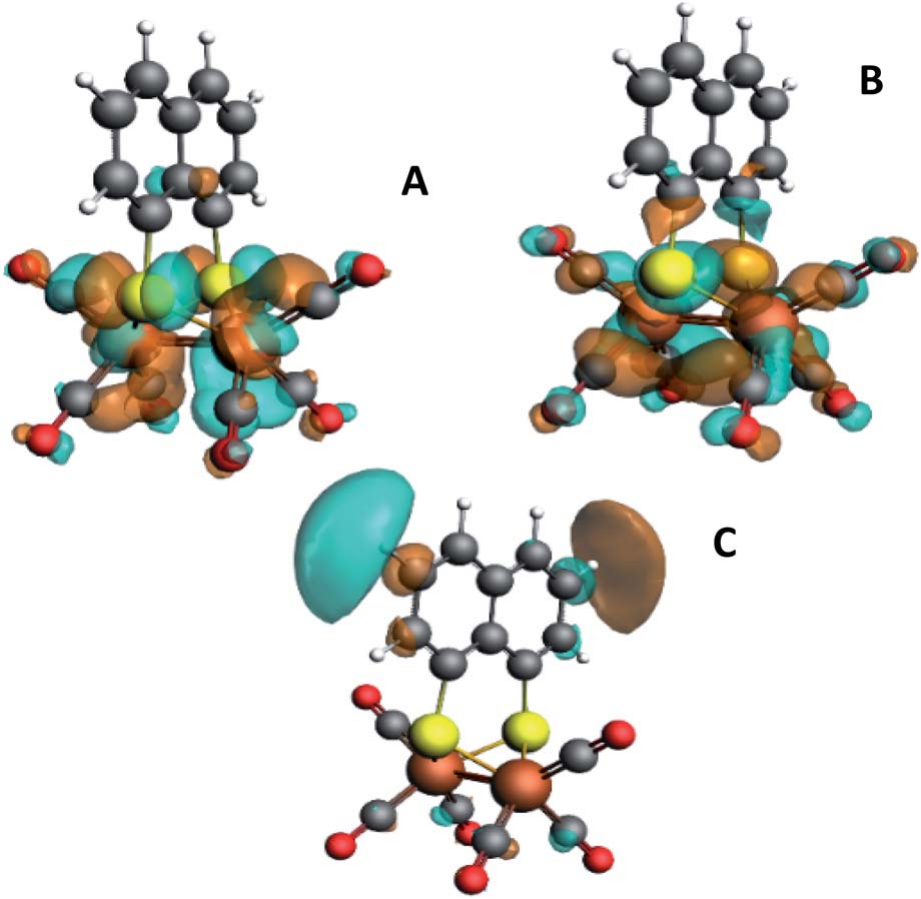
Isosurface plots of the unoccupied orbitals probed by the pre-edge transitions as obtained from TD-DFT calculations with B3LYP/QZ4P, for the major contributions to the pre-edge peaks of [2].

So far, EXAFS analysis showed that structural changes under reductive conditions are mainly observed in the iron–iron bond distance for [2], whereas [3]^1−^ showed no bond elongation between both iron atoms. Next to revealing additional structural information, Fe K-edge XANES also visualizes the electronic processes. The XANES data of [1], [2] and [3] in acetonitrile are shown in Fig. 2. All precursors have an absorption edge at ∼7125 eV. These are commonly observed for Fe^1^–Fe^1^ carbonyl ligated complexes,^30^ attributed to the strong π-accepting nature of the carbonyls.^31^ The shape and position of all three spectra are almost identical and supports a similar electronic structure and coordination environment near the absorbing iron atom. The position and intensity of the spectra in the pre-edge region (7112–7118 eV) are indicative of five coordinate iron species.^32^ The TD-DFT calculated pre-edge of [2] and [3] are both shown in Fig. 3. The peak at the start of the calculated spectrum is split in two transitions. The first transition, (7112.5 eV) only consists of quadrupole contributions. The five iron d-orbitals combine with the carbonyl σ and π*-orbital combinations, where the d_*xz*_- and d_*yz*_ orbitals interact only with the π*-orbitals (Fig. 2B peak A). The second transition (7112.8 eV) consists mostly of sulfur σ* contributions, with some small carbonyl σ contributions in addition. The peak at higher energy (7115 eV) originates from transitions where d_*z*^2^_ orbitals interact mainly with carbonyl π*-orbitals (Fig. 2B peak B). The involvement of the ligand is observed strongest at higher energies (7117–7119 eV) and are again similar in case of [2] and [3] (Fig. 2B peak C).

The isosurface plots of (combination of) unoccupied orbitals discussed above are plotted in Fig. 3, which also include the iron orbital contributions. Although the rising edge features are simulated in accordance with the experimental data, the dipole-allowed transitions cannot be accurately described with TD-DFT and therefore not further explained in combination with TD-DFT results.

Small differences at the rising edge (∼7120 eV) and the increased intensity at the top of the pre-edge (∼7112 eV) for complex [3] are most likely due to an overall lower symmetry compared to [2], enhancing 1s to 4p transitions.^33^ When examining the reduction events at [2] (Fig. 2), a significant edge shift of −1.5 eV was observed. No direct changes in the ligand environment around Fe observed, hence the edge shift strongly suggests a reduction of the iron core.^34^ No blue shift of the edge position is observed after the second electron reduction. The anionic charge is likely more distributed over the conjugated ligand backbone in this case. These statements are clearly reflected by the TD-DFT calculated spectrum of [2]^2−^ (Fig. S5†). The first two peaks in the pre-edge region are separated to a greater extend compared to [2]. This peak splitting can be understood by looking at the corresponding orbital contributions. In here, the first few LUMOs have more 3d character and less character of the CO π* orbitals in the reduced complex [2]^2−^, which are now reflected at 7114 eV. Thus, the metal and corresponding Fe–CO orbitals are affected to a greater extend in [2]^2−^. This matches the blue shift of the white line, another strong indication of metal reduction. Looking at the reduction of [3], the first derivative of the Fe K edge indicates no reduction of the iron centre itself, therefore strongly suggesting a ligand based reduction. The hypothesis of the charge being more located on the ligand backbone was confirmed by our TD-DFT XANES calculations (Fig. S5†). This is in line with EXAFS analysis, where no structural changes around the metallic centre took place, in contrast to [1]^2−^ and [2]^2−^ and further supports both the experimental and computational data.

### 3.4 S K-edge XANES

**Fig. 4:**
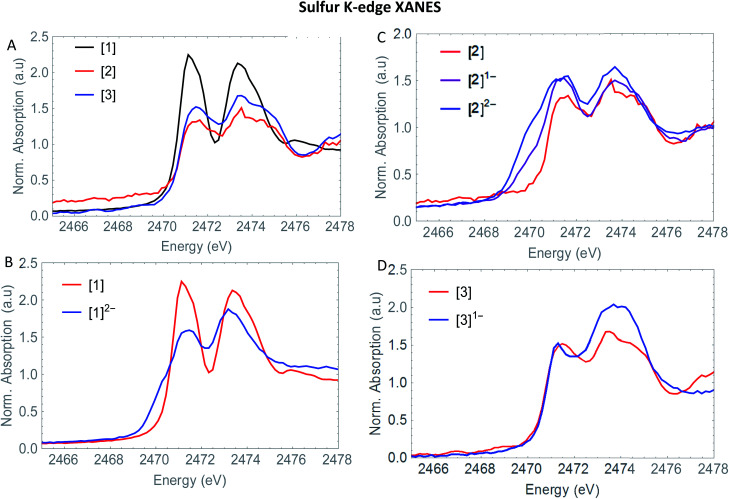
(A) Experimental S K-edge XANES of [1] (black) [2] (red) and [3] (blue). (B) Experimental S K-edge XANES spectrum of [2] (red), [2]^1−^ (purple) and [2]^2−^ blue. (C) Experimental S K-edge XANES spectrum of [2] (red), [2]^1−^ (purple) and [2]^2−^ (blue) and (D) experimental S K-edge XANES spectrum of [3] (red) and [3]^1−^ (blue).

The S XANES spectra in Fig. 4A define two separate features, at ∼2472 and at ∼2474 eV for all precursors [1], [2] and [3]. The first features at 2472 eV can be assigned to S 1s core electron excitations into vacant Fe–Fe, Fe–S mixed σ* and π* orbitals in addition to a limited set of Fe–CO π* orbitals that are symmetry allowed to mix with S 3p orbitals^30^ (Fig. 6). The features at 2474 eV emerge due to the excitation towards anti-bonding orbitals on the ligand backbone (Fig. 6).^35^ Comparing the three complexes (Fig. 4A), the naphthalene-bridged complexes exhibit a lower edge feature at 2474 eV. Comparing the calculated orbital compositions (Table S2†) reveals that [1] has the least (covalent) mixing between the ligand π* and sulfur orbitals, and the most iron character in the ligand π*-orbitals. The high intensity of both peaks in [1] are due to close lying high intensity transitions, whereas the broader intensity in [2] and [3] have additional transitions that are more spread out, giving rise to a lower and broader signal.^36^ By calculating the XANES spectrum of [1] using TD-DFT (Fig. 6A), the first feature at 2472 eV can be assigned to S 1s core electron excitations into the LUMO, mainly localized on the Fe_2_CO_6_ core with additional C-σ* mixing. Upon reduction of [1] with 2 eq. of 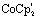 the large pre-edge signal at ∼2472 eV reduces drastically, together with a growing inset at lower energies (2470 eV), hinting at reduction of the sulphur atom(s). Looking in more detail, TD-DFT indeed shows a similar trend where the LUMO shifts to lower energy, which is still mainly located on the Fe_2_CO_6_ moiety accompanied by mixing of π* ligand backbone orbitals (Fig. 6A peak C). The additional lowering and blue shift of the second peak (B → D) is due to symmetry breakage and really emphasizes the formation of the open di-anionic species, explained by the excitation towards more unoccupied orbitals, spread out over a wider energy range.^30^

Adding one equivalent of reductant to [2] shows an intense pre-edge feature to arise, which further increases in intensity upon a second reduction (Fig. 4C). The pre-edge feature can be assigned to the excitation of 1s to the LUMO (Fig. 6B), visualizing a strong reduction on the iron core. This is accompanied by a minor blue shift of the rising edge (2473.9 eV). The shift shows the lowering of the ionization threshold, mainly caused by (indirect) reduction of sulfur species. The increased sulfide character delocalized between the two sub-clusters could be explained by partial spin density localized on the ligand backbone from the iron core.^37^ The increased absorption at 2474 eV which is also reflected in the TD-DFT calculation supports the spin density distribution towards the ligand backbone.

**Fig. 5:**
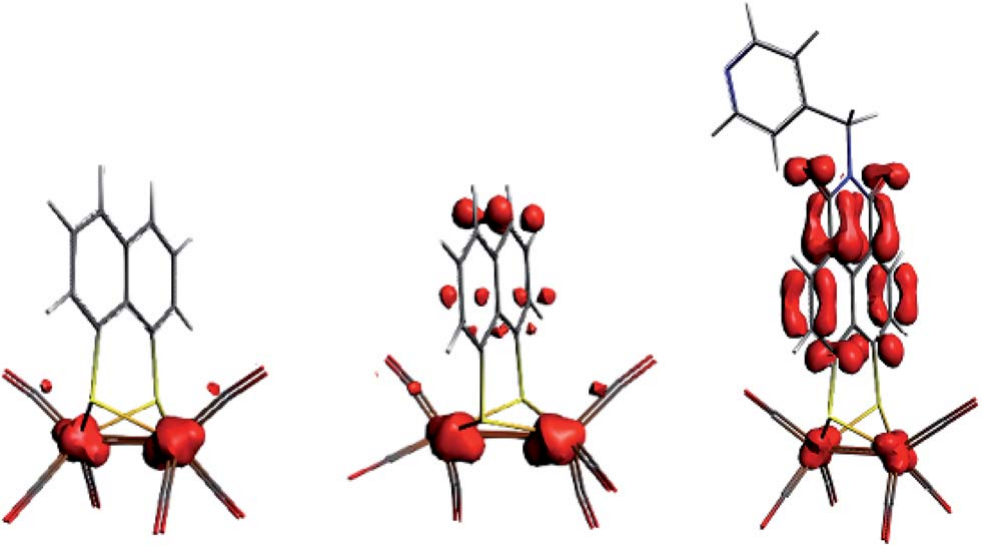
Spin density plot (BP86, dispersion Grimme3, TZ2P) for [2]^1−^, [2]^2−^ and [3]^1−^.

**Fig. 6:**
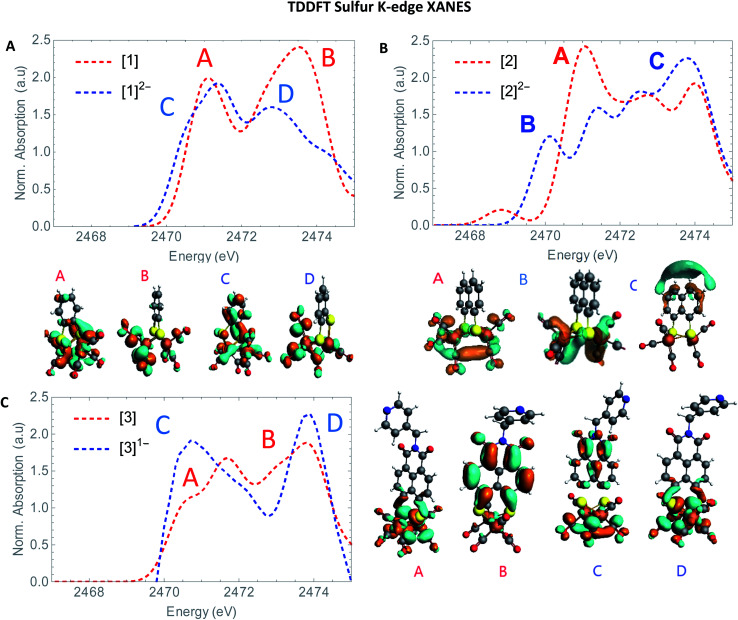
TD-DFT calculated S K-edge XANES (A) of [1] (red) and [1]^2−^ (blue). (B) [2] (red) and [2]^2−^ blue. (C) [3] (red) and [3]^1−^ (blue) with most contributing molecular orbitals.

Both Fe and S K-edge have shown us the reduction process to take place at the metallic site. This result is in line with the observed Fe–Fe bond elongation in our EXAFS analysis. Overall, the spin density distribution in [2]^1−^ and [2]^2−^ have experimentally been examined in solution and match the trend observed computationally. Fig. 5 shows the calculated spin density of [2]^1−^ and [2]^2−^ to mainly reside on the iron core.In comparison, reduction of [3] only shows a minor shift (∼−0.1 eV) of the edge at 2471 eV, together with a strong increase in absorption at 2474 eV. No changes in the pre-edge region, are a strong indication of the reduction event to take place further away from the Fe_2_S_2_ core, only affecting the transitions towards the ligand backbone, observed by increased absorption at 2474 eV and not the Fe–Fe or Fe–CO π* orbitals.^30^ The TD-DFT calculated spectrum of [3]^1−^ (Fig. 6C) points out unambiguously that the metallic part (2470 eV) is not affected whereas the ligand part (reflected at 2474 eV) is. These observations are in line with the EXAFS analysis of [3]^1−^ where no structural changes were observed. Furthermore, the calculated spin density of [3]^1−^, is mainly located on the NMI ligand (Fig. 5), further supporting our findings.

**Fig. 7:**
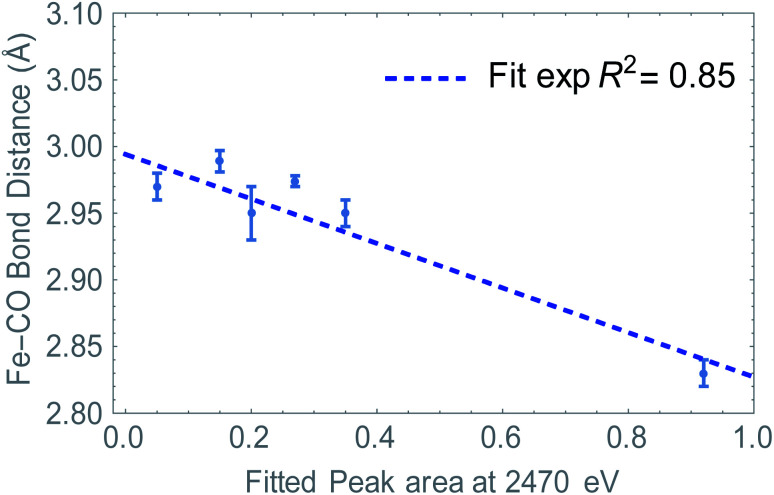
The absorption values at 2470 eV determined by pseudo-Voight peak fitting after abstraction of the two main peaks. The S-XANES data are plotted as a function of Fe–CO bond lengths. (Blue) Experimentally derived values *via* Fe–K edge EXAFS analysis.

Summarizing, we have determined that the distribution of the electron density is directly probed in S K-edge XANES. At the same time, we see a change in Fe–CO distance depending on ligand and/or upon reduction. Since the LUMO resides mostly on the Fe_2_S_2_CO_6_ fragment, we hypothesized that the S K edge and Fe–CO bond distance are a measure of the same phenomenon. Plotting the absorption at 2470 eV (peak area, see Fig. S7†) against the Fe–CO bond distance, we see that indeed a linear correlation was found (Fig. 7). This means that in this case, the S K-edge pre-edge region can be used to visualize the Fe–CO bond distance, as a function of ligand and/or reduction.

A direct link towards catalytic activity is difficult to establish, especially when structures undergo structural rearrangements, as observed for [1].^4^ These observations do however support the earlier proposed hypothesis that the LUMO should be primarily metal centered,^38^ explaining why [2]^6^ is more active than [3]^10^ (comparing the TOF).^39–41^ It should be noted that the rigidity of these naphthalene systems, together with their stabilizing charge effect results in more stable reduced species compared to alkyl-thiolate linkers.^42^

## 4. Conclusions

The combination of soft (S K edge) and hard (Fe K edge) X-ray spectroscopy enabled the full characterization of all parts of different hydrogenase catalytic systems. The S XANES spectra clearly showed electronic and structural changes due to high experimental energy resolution. This allowed us to differentiate between metallic reduction in case of [1] and [2] and ligand reduction in case of [3]. The bond breakage of Fe–S in [1]^2−^ has now been fully characterized by additional S K-edge XANES data. In here, TD-DFT calculations showed how the molecular orbitals are reorganized, explaining the blue shift of the edge in combination with damped peaks. Next to the shift or lack of shift in the edge position, additional evolution of pre-edge features in [2] were clearly assigned by TD-DFT. A linear correlation between the peak area of the pre-edge in S K-edge XANES and the EXAFS determined Fe–CO bond distance reflects the changed spin density in the systems, affecting both S electronics and Fe–CO bond length in equal measure. A satisfactory derived elongation of the Fe–Fe bond in [2]^2−^ with no additional Fe–Fe scattering paths at longer distances show the Fe_2_S_2_ core to stay intact. No Fe–Fe bond elongation was observed *via* EXAFS analysis in [3]^1−^ underpinning the non-innocence of the NMI group. This study shows that a combination of soft and hard X-rays is a powerful tool to identify redistribution of charge in the ligand during chemical processes. These results are a good starting point for operando (soft) X-ray catalysis to study the ligand under catalytic conditions, for example, electrochemical formation of H_2_.

## Conflicts of interest

There are no conflicts to declare.

## Supplementary Material

RA-010-C9RA08903A-s001
