# Supplementary material for: Electronic characterization of redox (non)-innocent Fe2S2 reference systems: a multi K-edge X-ray spectroscopic study
Source: RSC Adv. 2020 Jan 2;10(2):729–38. doi: 10.1039/c9ra08903a (PMC9048190; doi:10.1039/c9ra08903a)
Supplement: RA-010-C9RA08903A-s001 [file RA-010-C9RA08903A-s001.pdf]

Electronic Supplementary Material (ESI) for RSC Advances.
This journal is © The Royal Society of Chemistry 2019

Supporting Information

Electronic Characterization of Redox (Non)-Innocent Fe_2S_2 Reference Systems: Multi edge X-ray Spectroscopic study

J.P.H. Oudsen[a], B. Venderbosch[a], T.J. Korstanje [a] and M. Tromp[b]

[a.] Sustainable Materials and Characterization, van ’t Hoff Institute for Molecular Sciences, University of Amsterdam, Science Park 904, 1098 XH Amsterdam, The Netherlands.

[b.] Materials Chemistry, Zernike Institute for Advanced Materials, Nijenborgh 4, 9747AG Groningen, the Netherlands.

1. IR Spectroscopy

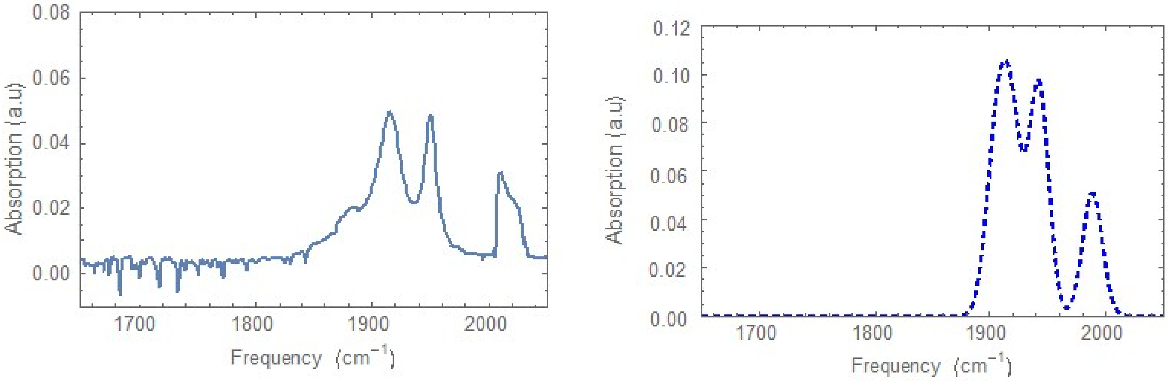

Figure S1. Absorbance difference IR SEC spectra recorded during the first electron reduction of 1.0 mM of [2] in Acetonitrile (0.1 M nBu_4NPF_6) within an OTTLE cell.

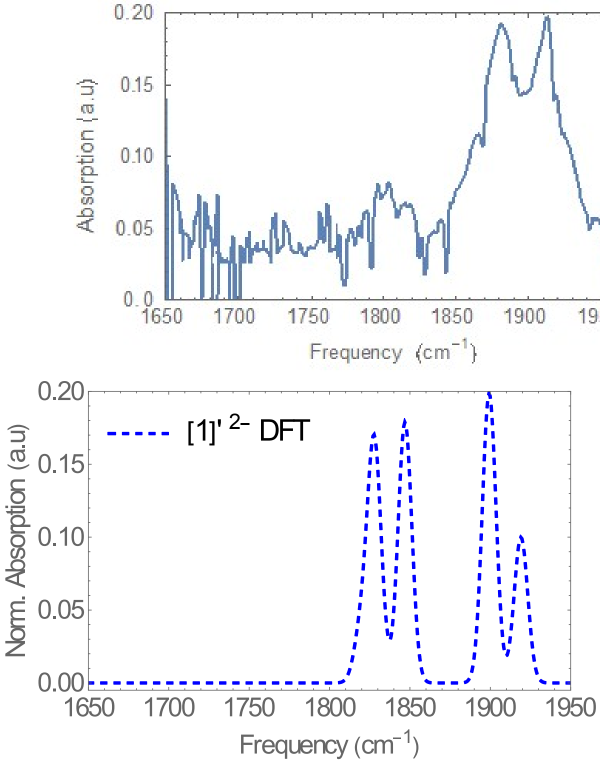

Figure S2. TDDFT calculated spectra of $[2]^{2-}$ (right) compared to the final spectrum in the spectro-electrochemistry measurement (left).

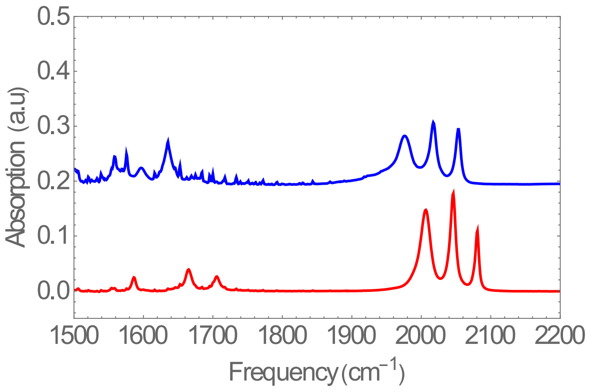

Figure S3. Steady state infrared spectrum of hydrogenase complex [3] (bottom red) and [3] + 1.05 eq. $CoCp_2$* (top blue).

2. XAS Analysis

Table S1. Fe K-edge EXAFS fitting parameters for $[1]^{1-}$ (DFT geometry optimized), where N = coordination number, σ^2 = Debye Waller factor [Å^{-2}], R = fitted bond length [Å].

Sample	*Shell*	*N*	σ^2	$R_{XRD/DFT}$ (Å)	R_{fit} (Å)
$[1]^{1-a}$	Fe - C	3	0.004(1)	1.80	1.806(8)
	Fe - S	2	0.0018(7)	2.33	2.27(1)
	Fe - Fe	1	0.007(5)	2.85	3.12(4)
	Fe - O	3	0.011(4)	2.93	2.95(1)
	Fe - CO	2	0.008(4)	2.93	2.95(1)
	Fe - CO	2	0.008(4)	2.93	2.95(1)
	Fe - COC	2	0.008(4)	2.93	2.95(1)

[a]k range = 3 – 10.5 Å, R range = 1 – 4 Å; k-weighted fit = 1,2,3 E_0 = 1.16* eV, S_0^2 = 0.90. R-factor fit: 0.015

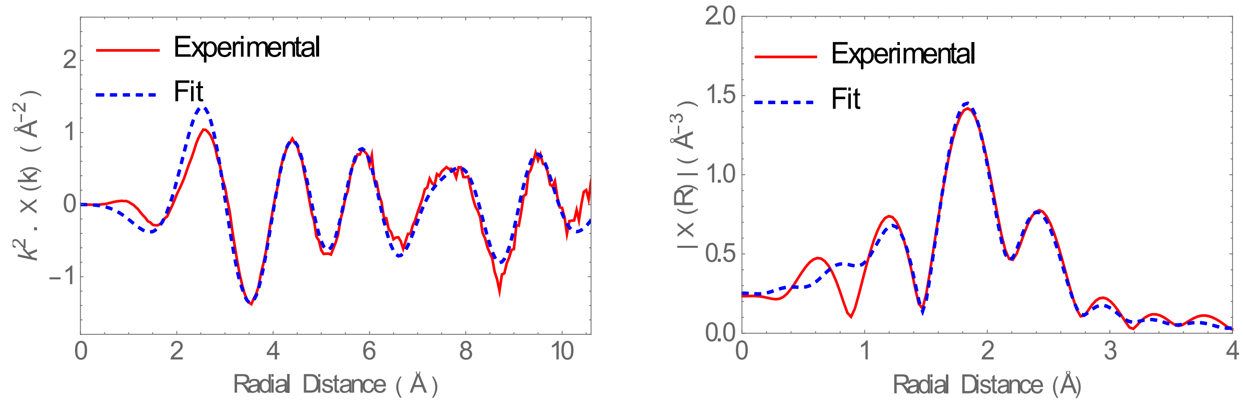

Figure S4. (Left) k^2-weighted Fe K-edge EXAFS data of *$[1]^{1-}$* and (right) k^2-weighted Fourier Transforms of the EXAFS data for 3 < k < 10.5 Å of *$[1]^{1-}$*. In all plots the data is represented by the solid lines (red), whereas the corresponding fits are the dotted lines (blue).

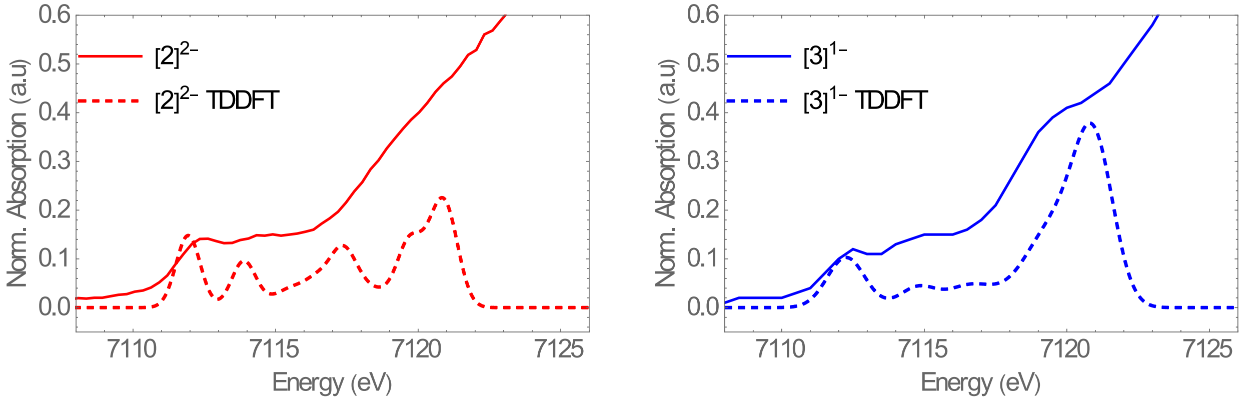

Figure S5. Experimental (solid) and computational (dashed) Fe K edge XANES spectrum of (left) *[2]*$^{2-}$ (red) and (right) *[3]*$^{1-}$ (blue).

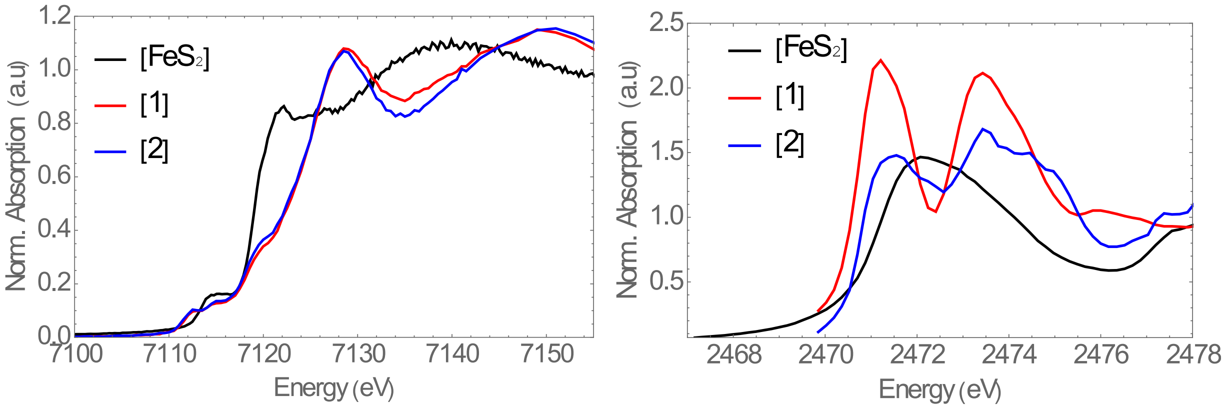

Figure S6. Experimental Fe K-edge (left) and Fe S K-edge (right) XANES spectrum of reference material FeS_2 and complexes *[1]* and *[2]*.

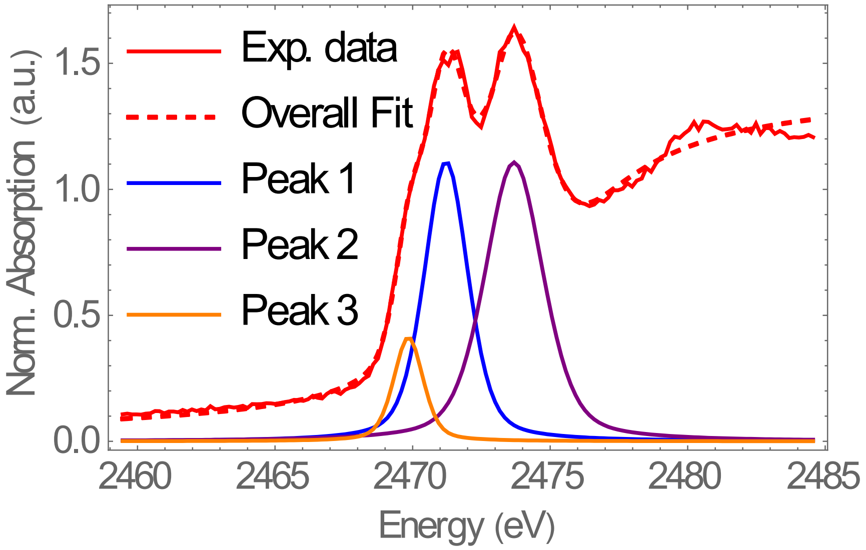

Figure S7. Example of pseudo-Voight peak fitting of experimental S K edge XANES data of $[1]^{2-}$.

3. Molecular Orbital analysis

Table S2. Percentage of Symmetrized Fragment orbitals in the Lowest Unoccupied (LUMO) and Highest Occupied Molecular Orbitals (HOMOs) of [1] – [3].

Compound	*Molecular Orbital*	*%Fe d_{z^2}*	*%Fe d_{xy}*	*%Fe d_{xz}*	*%Fe d_{yz}*	*%Fe $d_{x^2-y^2}$*	*%S P_x*	*%C* P_x*
[1]	HOMO	13	-	1	13	21	9	13
	HOMO-1	8	3	14	9	13	-	-
	HOMO-2	10	40	9	5	8	-	-
	LUMO	12	11	5	-	7	9	8
	LUMO+1	4	5	11	9	14	13	1
[2]	HOMO	9	-	20	-	20	6	-
	HOMO-1	-	3	-	3	-	5	60
	HOMO-2	-	8	-	16		52	-
	LUMO	6	-	18	-	20	11	5
	LUMO+1	-	5	-	10	-	-	56
[3]	HOMO	-	-	-	-	-	-	70**
	HOMO-1	12	-	17	-	15	-	-
	HOMO-2	34	-	20	-	23	7	-
	LUMO	-	4	-	8	-	-	-
	LUMO+1	6	-	18	-	20	11	3

***%C only involves the ligand backbone and not the carbonyl based orbitals.**

****%C also involves the %N in the ligand backbone.**

4. TDDFT input file

Example of a TDDFT input file for sulfur and iron K-edge XANES in case of ***[1]***.

```
#!/bin/bash

#SBATCH -N 1
#SBATCH -n 16
#SBATCH -t 80:00:00

module load pre2019
module load adf/2017.107
module load paffinity
module load openmpi/gnu


ulimit -s xxxxxxx


adf <<eor

ATOMS
1 Fe       2.232898000000       0.481404000000       3.539259000000
2 Fe       4.159515000000       1.340844000000       2.234532000000
3 S       2.551162000000       2.684641000000       3.087046000000
4 S       2.345244000000       0.379512000000       1.273579000000
5 C       2.749762000000       0.788558000000       5.214293000000
6 O       3.103996000000       1.011540000000       6.270994000000
7 C       2.656720000000      -1.253718000000       3.620011000000
8 O       2.911107000000      -2.361243000000       3.651158000000
9 C       5.307832000000       1.910850000000       3.473503000000
10 O       6.038188000000       2.241631000000       4.270644000000
11 C       5.121833000000      -0.165390000000       2.066106000000
12 O       5.750415000000      -1.114908000000       2.014194000000
13 C       0.446337000000       0.379512000000       3.784977000000
14 O      -0.665715000000       0.286480000000       3.940713000000
15 C       4.767849000000       2.281502000000       0.828288000000
16 O       5.128809000000       2.848554000000      -0.087674000000
17 C       1.481689000000       2.895809000000       1.685416000000
18 C       1.374312000000       1.806004000000       0.844439000000
19 C       0.541434000000       1.869502000000      -0.258407000000
20 C      -0.166730000000       3.018375000000      -0.496050000000
21 C      -0.067766000000       4.086029000000       0.323009000000
22 C       0.770799000000       4.038775000000       1.448927000000
23 H       0.463891000000       1.141489000000      -0.831749000000
24 H      -0.726934000000       3.064152000000      -1.236664000000
25 H      -0.556324000000       4.856866000000       0.140740000000
26 H       0.841182000000       4.768264000000       2.021115000000
END
```

```
SYMMETRY NOSYM

CHARGE -2.0 0.0


BASIS
type QZ4P
core None
createoutput None
END

XC
Hybrid B3LYP
END


EXCITATIONS
Davidson
ONLYSING
lowest 50
NTO
XAS
ALLXASMOMENTS
ALLXASQUADRUPOLE
END

MODIFYEXCITATION
UseOccupied
A 3 %this number can be changed to e.g. 1 to use Fe instead of S 1s)
    SubEnd
END

NumericalQuality VeryGood

NOPRINT LOGFILE

eor
```

5. DFT reaction coordinates

All coordinates are shown for OPBE basis set in the gas phase.

[2]

Fe	0.56705532	4.74134134	3.25529097
Fe	2.90108696	4.71416953	2.24246813
S	2.18785609	3.26463739	3.83544929
S	2.15787782	6.30215298	3.68150647
C	2.80901121	5.92536168	0.93479560
C	-0.42865746	4.82539257	4.73142470
O	-1.06510519	4.88289133	5.69384376
O	-0.82688329	2.55942125	1.86261745
O	-0.83514159	6.71444855	1.58653286
O	5.79900270	4.73661024	2.72628486
O	2.73023128	2.53751623	0.27374284
O	2.75222121	6.69651154	0.07692385
C	2.79861678	3.38473709	1.05557220
C	-0.27300094	3.40984238	2.41410447
C	2.88984193	3.61151606	5.43413965
C	3.19442474	2.46862506	6.16270562
H	2.99122166	1.49270113	5.72221442
C	3.75691797	2.55111865	7.45061119
H	3.98496687	1.63668854	7.99675063
C	4.01381575	3.78437318	8.00654539
C	3.71815865	4.97740145	7.29765493
C	3.98792845	6.24142289	7.88303851
C	3.70554487	7.40697408	7.20656732
H	3.91369487	8.37614526	7.65805331
C	3.14278447	7.34815218	5.91744565
H	2.91961861	8.27056607	5.38162909
C	2.86322579	6.13190633	5.30723267
C	3.14195049	4.90502906	5.97822335
H	4.44954380	3.86336538	9.00295392
H	4.42420365	6.27189911	8.88190104
C	4.66064341	4.72901989	2.52878933
C	-0.27941528	5.94795165	2.24824756

[2]$^{1-}$

Fe	0.42592518	4.74855940	3.34435033
Fe	3.06378157	4.70506754	2.19865136
S	2.14795714	3.24714109	3.75767667
S	2.12900090	6.31403722	3.59338767
C	3.00366634	5.88110944	0.88647193
C	-0.42210608	4.81149669	4.92242396
O	-1.10626919	4.84233369	5.86735015
O	-1.01272621	2.61434552	1.94315750
O	-1.07866994	6.72575028	1.79243222

O	5.95373177	4.74055626	2.80788072
O	2.98813155	2.54424979	0.22053982
O	2.99376149	6.65465033	0.01284274
C	2.99864528	3.40092475	1.01284670
C	-0.42394488	3.46016134	2.49030346
C	2.84849042	3.60290958	5.35452971
C	3.15429720	2.47068912	6.09899184
H	2.94820960	1.49328235	5.66151905
C	3.71615423	2.55740664	7.38725600
H	3.94203369	1.64433881	7.93980254
C	3.97489075	3.79261621	7.93743686
C	3.68118367	4.98066668	7.21866210
C	3.95705087	6.24198985	7.80750510
C	3.68071603	7.40804362	7.12917392
H	3.89265704	8.37782972	7.58153235
C	3.11974584	7.34953708	5.83922318
H	2.90088988	8.27206767	5.30048002
C	2.83087041	6.14059791	5.21827280
C	3.10522578	4.90608588	5.89625667
H	4.40983766	3.88042072	8.93477315
H	4.39237699	6.26676758	8.80820896
C	4.79619542	4.73256059	2.65868609
C	-0.46476779	5.94115108	2.39970269

[2]$^{2-}$

Fe	0.18860246	4.88608580	3.51889928
Fe	3.35939968	4.83852779	2.14263302
S	1.99149244	3.31101808	3.38386039
S	2.08861025	6.26574489	3.50695522
C	2.77835698	5.59217963	0.66046157
C	-0.38795761	5.42929814	5.08783151
O	-0.91789864	5.81660514	6.07214184
O	-1.76139244	2.71592615	3.44930761
O	-1.08245778	6.13707616	1.18896594
O	6.00855905	5.69353646	3.07098425
O	4.56443194	2.62167303	0.67631574
O	2.56603765	6.10604425	-0.38142051
C	4.08199551	3.52126373	1.26550788
C	-0.97861940	3.59672206	3.47721747
C	2.69636029	3.42210787	5.00782428
C	2.95704438	2.21414827	5.65279870
H	2.71331680	1.29255327	5.12221273
C	3.51461398	2.15546257	6.94389695
H	3.69957137	1.18237166	7.40645825
C	3.82368340	3.31851186	7.61773822
C	3.58097751	4.58137559	7.01072801
C	3.90440020	5.77337075	7.71716855
C	3.67888274	7.00733107	7.15125133
H	3.92832398	7.92158377	7.69528239
C	3.12134027	7.09439873	5.85909472
H	2.93988772	8.06970863	5.40591933

C	2.78795546	5.96197075	5.12972176
C	3.01056622	4.65690127	5.68972083
H	4.25669747	3.29250148	8.62071471
H	4.33555461	5.68524884	8.71777193
C	4.91540066	5.33764008	2.79165568
C	-0.47663633	5.62823544	2.06568743

[3]

Fe	0.53892931	4.74325707	3.31125545
Fe	2.77982941	4.76349552	2.10777859
S	2.21541681	3.26914428	3.71972899
S	2.14257714	6.31630954	3.63591715
O	5.42253461	2.74222302	9.63622537
O	5.32879625	7.32046285	9.50138163
O	-0.89638957	4.77381197	5.87593074
O	-0.93072293	2.59471991	1.94650456
O	-1.03370504	6.74297231	1.83916969
O	5.70902168	4.85115357	2.33310905
O	2.51214885	2.61804529	0.11672338
O	2.38958417	6.76996185	-0.00438835
N	5.34935803	5.03139227	9.59144670
N	10.36076309	5.13663093	10.26059462
C	3.04082709	3.59406766	5.26256292
C	3.41626933	2.44016272	5.94353215
H	3.18797951	1.46939963	5.50395694
C	4.07984186	2.50450397	7.17553118
H	4.37294616	1.59788762	7.70308516
C	4.37466060	3.73273032	7.74165246
C	4.00722015	4.92990561	7.07647310
C	4.32145132	6.17811092	7.67149530
C	3.97297253	7.35783598	7.03687787
H	4.22600088	8.30510715	7.51100449
C	3.30692446	7.32246294	5.80507670
H	3.03506354	8.25554614	5.31196301
C	2.98332591	6.11621324	5.19154018
C	3.32646572	4.87875523	5.81134824
C	5.08255495	3.76364768	9.04766689
C	5.03051754	6.25294716	8.97513089
C	6.07124258	5.08341536	10.88354701
H	5.76779423	4.19901583	11.45181154
H	5.73775078	5.99099327	11.39566604
C	7.57063890	5.10231391	10.68051257
C	8.26299009	6.30778424	10.52364005
H	7.72823012	7.25680832	10.55084338
C	9.64371258	6.26975382	10.31865229
H	10.20511605	7.19954025	10.19577743
C	9.68572954	3.98667919	10.41076013
H	10.28051301	3.07088130	10.36203109
C	8.30666500	3.91457779	10.62000334
H	7.80770797	2.95138673	10.72305219

C	-0.33890771	4.76203109	4.86496432
C	-0.34855579	3.43105914	2.48822770
C	-0.41166411	5.96532379	2.42240724
C	4.55916766	4.81634050	2.23713911
C	2.61703706	3.45405339	0.90529949
C	2.54279177	5.98937763	0.83166860

[3]$^{1-}$

Fe	0.51502945	4.76192609	3.29463686
Fe	2.75058486	4.69346844	2.07499152
S	2.17468341	3.25558577	3.76418598
S	2.19281316	6.28673460	3.62340502
O	5.40931542	2.72726260	9.65645068
O	5.42300292	7.31645382	9.45471326
O	-0.93410227	4.93067215	5.83970510
O	-1.02858839	2.60655395	2.01887946
O	-0.97155862	6.77342640	1.74517970
O	5.67782962	4.65063603	2.25648091
O	2.34655315	2.53262144	0.11933237
O	2.43785465	6.69538755	-0.05891819
N	5.38398462	5.02302988	9.53720016
N	10.42250992	5.33735044	10.53481653
C	3.01264734	3.57747768	5.27271744
C	3.36758250	2.40677017	5.97784603
H	3.10506597	1.44032700	5.54644342
C	4.03318983	2.47084423	7.18804348
H	4.30930833	1.56894801	7.73280953
C	4.37079850	3.71452928	7.74656847
C	4.03262450	4.91690349	7.06711710
C	4.38040692	6.17304698	7.63459686
C	4.05225126	7.36367680	6.96638541
H	4.33577643	8.30872838	7.42784098
C	3.38750745	7.32317889	5.75458275
H	3.13316705	8.24854066	5.23687229
C	3.02385463	6.09627818	5.15801648
C	3.33851008	4.86210507	5.80001228
C	5.07560843	3.74195395	9.02532924
C	5.08503093	6.25458040	8.91123683
C	6.09178655	5.08583720	10.81001308
H	5.84582525	4.16663216	11.35755988
H	5.72059785	5.95928798	11.35935671
C	7.59580605	5.18546550	10.68275826
C	8.37331424	5.48714507	11.80710445
H	7.90090405	5.67878173	12.77269514
C	9.76019797	5.54957459	11.68445076
H	10.37661947	5.78803861	12.55639166
C	9.66726637	5.05073945	9.46234888
H	10.20824018	4.87982324	8.52740835
C	8.27410665	4.96360536	9.48348460
H	7.72497882	4.72781703	8.57246041

C	-0.35806157	4.86203289	4.83590422
C	-0.40173037	3.43827808	2.53012184
C	-0.36794210	6.00108701	2.36581129
C	4.52086918	4.66852963	2.18567116
C	2.50328899	3.36390955	0.91341801
C	2.55664278	5.92722202	0.80237452
